# Dual Invasive Fungal Infections After Kidney Transplantation: A Case Report

**DOI:** 10.3390/jof12080621

**Published:** 2026-08-19

**Authors:** Layan Akkielah, Ahmed Bishara, Leigh J. Sowerby, John Johnson, Matthew A. Weir, Michael Chiu, Laila Alshafai, Michael Silverman, Mohammad Reza Rahimi Shahmirzadi

**Affiliations:** 1 Department of Medicine and Infectious Diseases, Western University, London Health Sciences Centre, London, ON N6A 3K7, Canada; layan.akkielah@lhsc.on.ca (L.A.); ahmed.bishara@lhsc.on.ca (A.B.); michael.silverman@lhsc.on.ca (M.S.); 2 Department of Otolaryngology-Head and Neck Surgery, Western University, London, ON N6A 3K7, Canada; leigh.sowerby@lhsc.on.ca; 3 Department of Medicine, Division of Nephrology, Schulich School of Medicine and Dentistry, Western University, London, ON N6A 3K7, Canada; john.johnson@lhsc.on.ca (J.J.); matthew.weir@lhsc.on.ca (M.A.W.); michael.chiu@lhsc.on.ca (M.C.); 4 Department of Epidemiology and Biostatistics, Western University, London, ON N6A 3K7, Canada; 5 Division of Neuroradiology, Diagnostic Neuroradiologist, Head and Neck Imaging, Mount Sinai Hospital, University Health Network, Toronto, ON M5G 1X5, Canada; laila.alshafai@uhn.ca

**Keywords:** kidney transplant, donor-derived infections, mucormycosis, *Candida albicans*

## Abstract

Donor-derived infections (DDIs), particularly fungal DDIs, are uncommon but serious complications of solid organ transplantation. We report a case of a 52-year-old woman who underwent deceased donor kidney transplantation complicated by probable donor-derived *Candida albicans* candidemia with native aortic valve endocarditis, managed medically with prolonged echinocandin therapy followed by suppressive fluconazole. Approximately 14 months post-transplant, she developed progressive rhino-orbital mucormycosis due to *Rhizopus oryzae*, requiring extensive surgical debridement and prolonged antifungal therapy. Initial treatment with liposomal amphotericin B was limited by nephrotoxicity, prompting transition to isavuconazole for long-term management. Immunosuppression was discontinued to control infection, resulting in graft failure. This case illustrates the complex interplay between donor-derived infection, antifungal exposure, and immunosuppression in transplant recipients. It highlights the potential contribution of antifungal selective pressure to breakthrough mold infections and underscores the importance of early recognition, aggressive multidisciplinary management, and individualized antifungal strategies in this high-risk population.

## 1. Introduction

Invasive fungal infections remain a significant cause of morbidity and mortality in solid organ transplant recipients, driven by the cumulative effects of immunosuppression, comorbidities, and healthcare-associated exposures [1,2]. Donor-derived infections represent a rare but clinically important subset, often presenting early after transplantation and posing unique diagnostic and therapeutic challenges. While bacterial and viral pathogens predominate, fungal donor-derived infections are less common but may result in severe invasive disease with high associated mortality [3,4]. The occurrence of multiple invasive fungal infections within the same transplant recipient is uncommon and reflects the complex interplay between host factors, antimicrobial exposure, and immunosuppression. Such cases highlight important challenges in diagnosis, antifungal selection, and long-term management in this high-risk population. Here, we describe a case of donor-derived *Candida albicans* infection complicated by subsequent invasive mucormycosis in a renal transplant recipient, illustrating the evolving infectious risks and management considerations encountered in transplant infectious diseases.

## 2. Case Report

**Figure 1:**
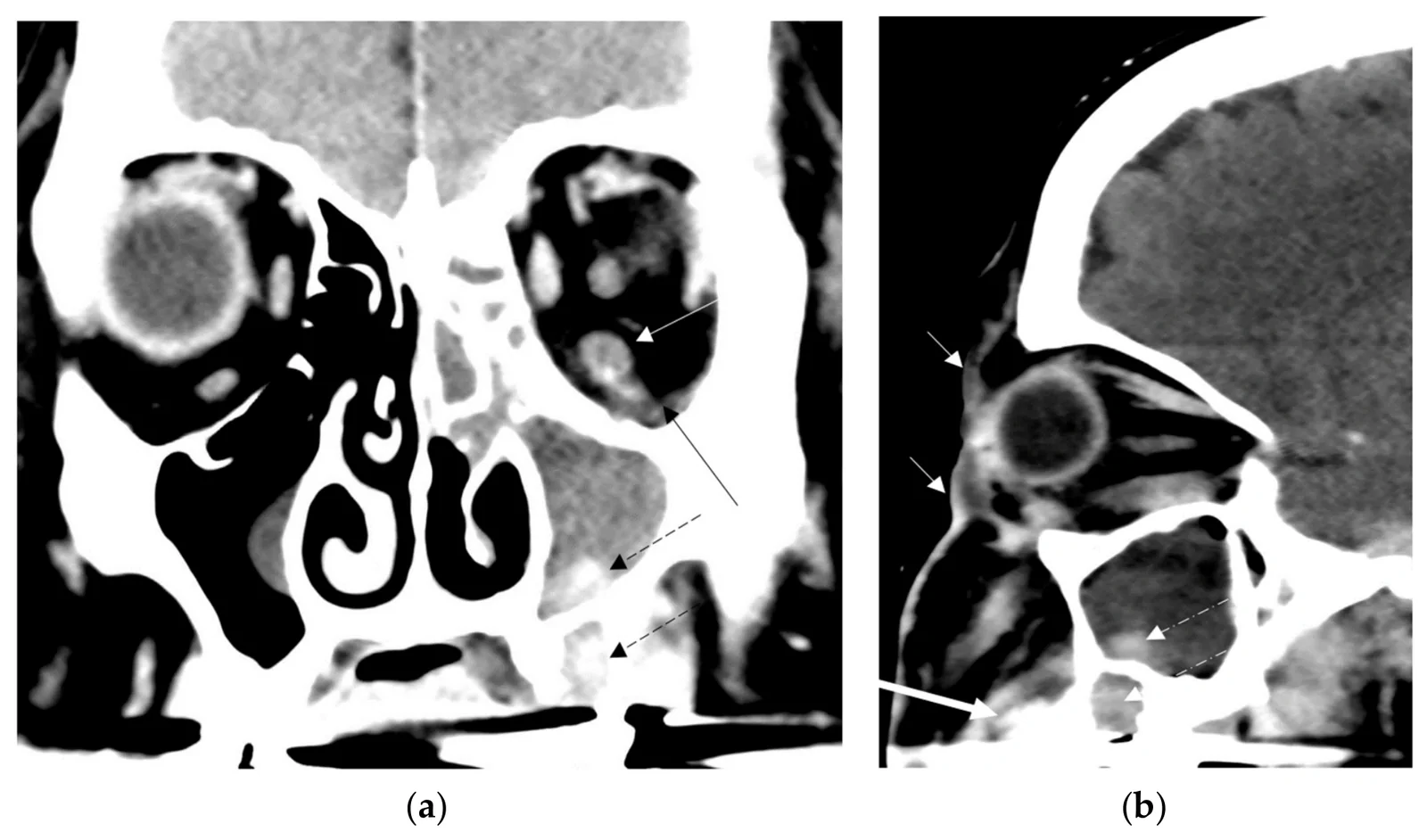
(**a**) Coronal reformat of CT head post-contrast demonstrating diffuse fat stranding in the left post-septal orbital space (black arrow) with slight enlargement of the extraocular muscles (white arrow). There is diffuse opacification of the left sides’ sinuses—in the floor of the left maxillary sinus with underlying odontogenic cyst (black dashed arrows). (**b**) Sagittal reformat of CT head post-contrast on the left side demonstrating diffuse fat stranding in the left pre-septal periorbital space (white short arrows) with slight enlargement of the extraocular muscles—in the floor of the left maxillary sinus with underlying odontogenic cyst (white dashed arrows) and abscess in the adjacent gingiva (white arrow).

**Figure 2:**
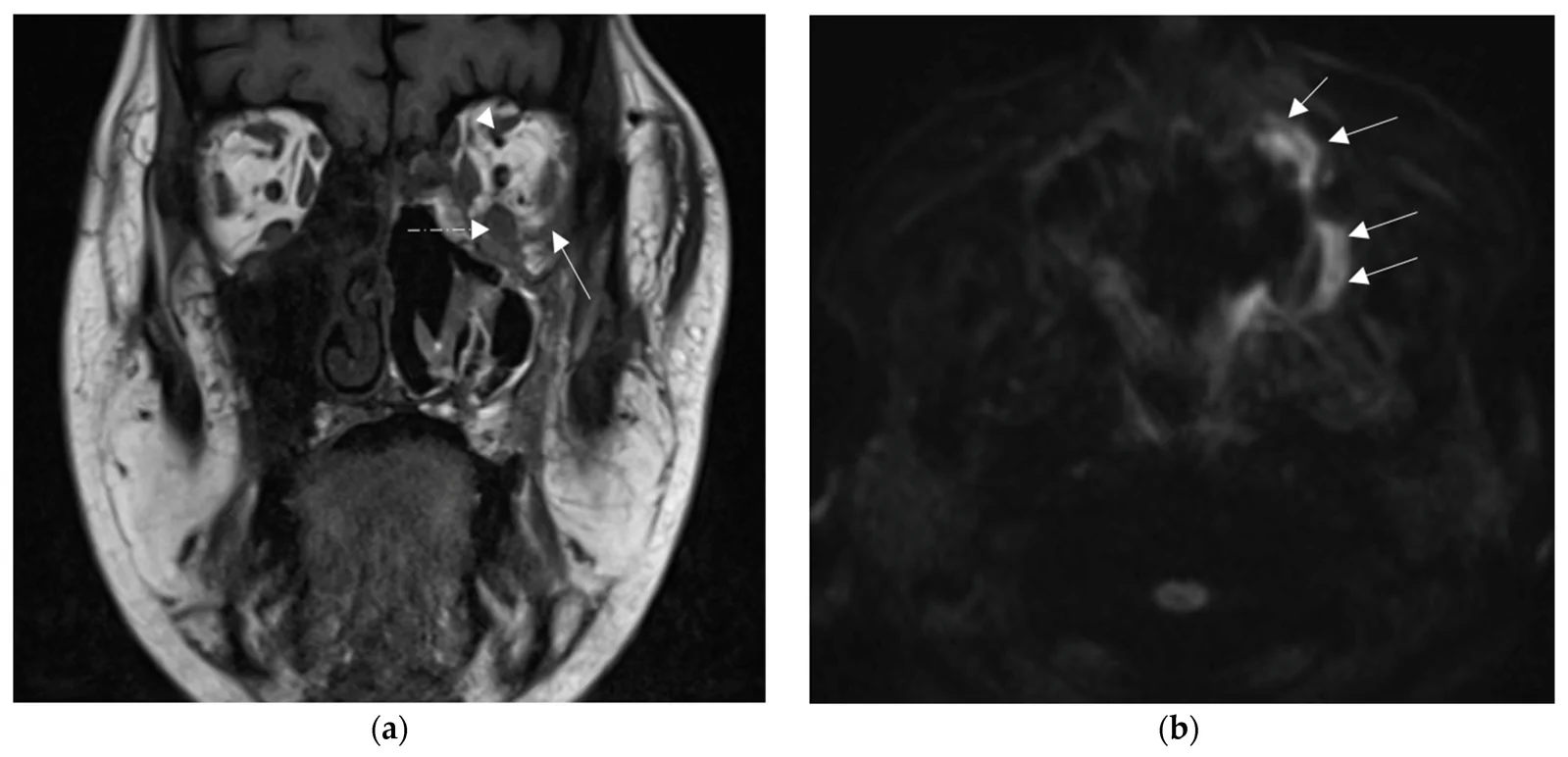
(**a**) Coronal T1 non-fat-saturated pre-gadolinium injection MRI sequence of the head. There has been interval left-sided endoscopic sinuonasal surgery for evacuation of the sinusitis. Persistent diffuse fat stranding in the left post-septal orbital space (white arrow) with slight enlargement of the extraocular muscles (white dashed arrow). The left superior ophthalmic vein is not dilated (white arrow head). (**b**) Axial diffusion weight sequence of the brain at the level of the floor of the maxillary sinuses. There is diffusion restriction in the outer gingiva (white arrows) in keeping with abscess (ADC map isnot shon but demonstrated low signal intensity concluding the positive restriction).

**Figure 3:**
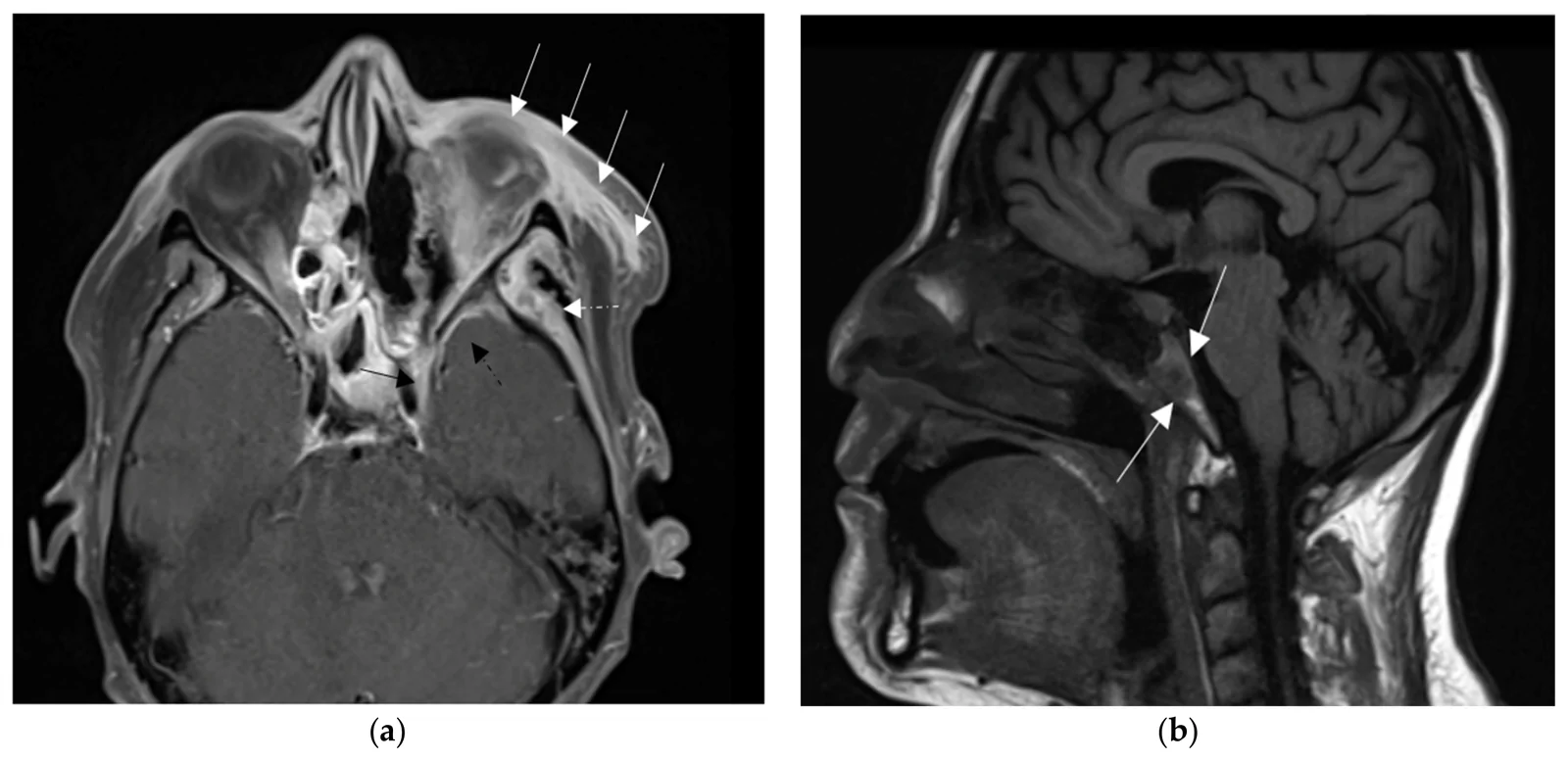
(**a**) Axial T1 fat-saturated post-gadolinium injection MRI sequence of the head. There is diffuse cellulitis of the left pre-septal periorbital soft tissue (white arrows) and diffuse myositis in the left upper lateral face and left suprazygomatic muscle (dashed white arrow). There is subtle increased enhancement of the left cavernous sinus (black arrow) and subtle pachymeningeal enhancement (black dashed arrow). (**b**) Sagittal T1 non-fat-saturated pre-gadolinium injection MRI sequence of the head and neck demonstrating low signal intensity in the clivus with thickening of the nasopharynx concerning for early acute osteomyelitis (white arrows).

**Figure 4:**
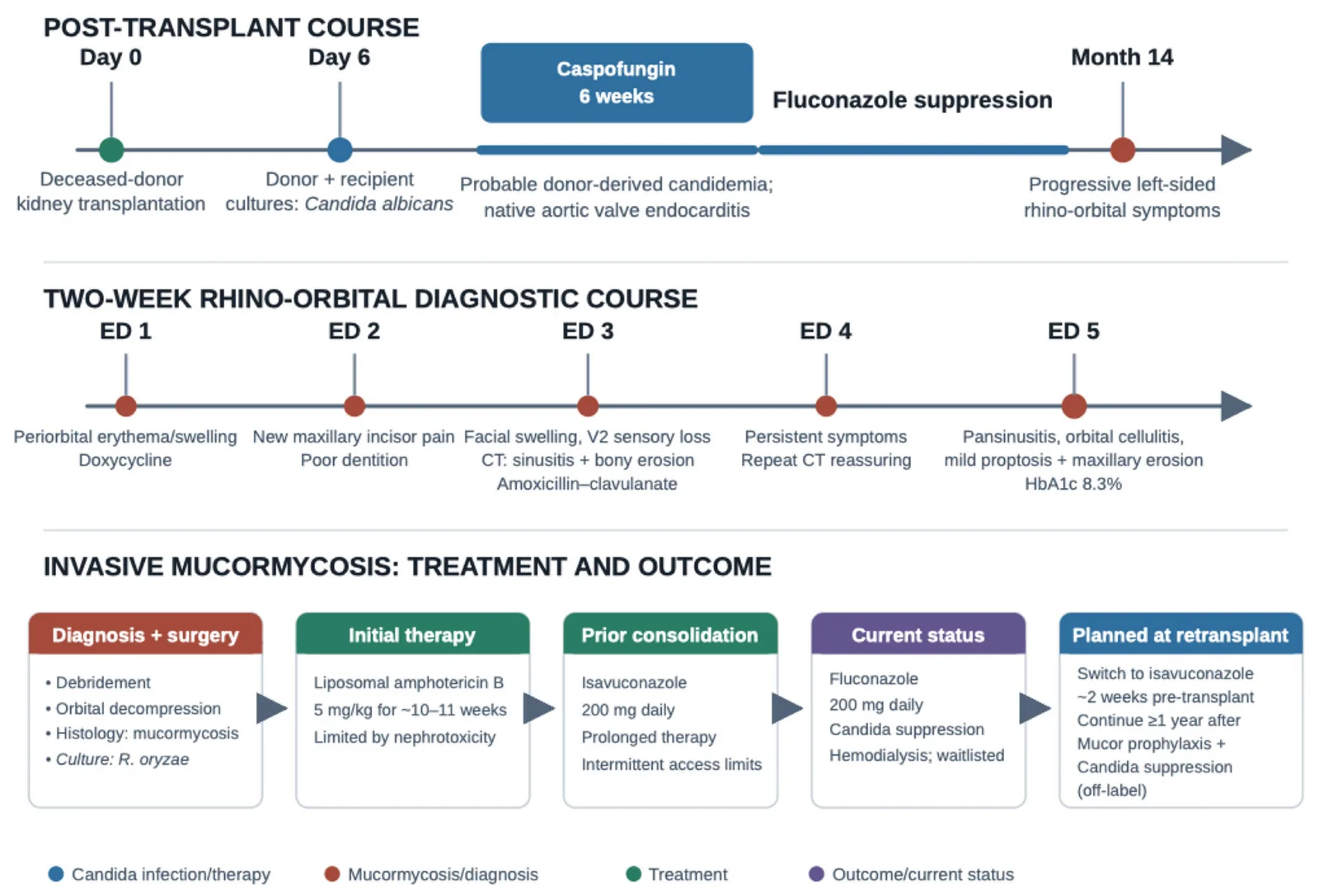
Clinical timeline of the two sequential invasive fungal infections. Top (post-transplant course): deceased-donor kidney transplantation (Day 0), donor-derived Candida albicans candidemia with native aortic valve endocarditis (Day 6; caspofungin for 6 weeks followed by fluconazole suppression), and the onset of rhino-orbital symptoms at month 14. Middle (two-week rhino-orbital diagnostic course): the five emergency department (ED) encounters preceding diagnosis. Bottom (invasive mucormycosis): diagnosis and surgery, initial and consolidation antifungal therapy, current status, and the planned peri-retransplant strategy. Colors indicate Candida infection/therapy, mucormycosis/diagnosis, treatment, and outcome/current status, as shown in the legend. CT, computed tomography; ED, emergency department; HbA1c, glycated hemoglobin; R. oryzae, Rhizopus oryzae.

A 52-year-old woman with end-stage renal disease (ESRD), her renal failure was presumed to be multifactorial, with acute tubular necrosis in the setting of septic shock, following an episode of severe pneumosepsis secondary to *Legionella pneumophila*, as the primary driver, compounded by a concurrent new diagnosis of diabetes mellitus. She was initially managed with intermittent hemodialysis, transitioned to peritoneal dialysis, and subsequently returned to hemodialysis due to technique failure. She ultimately underwent deceased donor kidney transplantation. The allograft was procured from a deceased donor classified as an expanded criteria donor (ECD) and donation after circulatory death (DCD). Induction immunosuppression consisted of rabbit anti-thymocyte globulin (rATG). Maintenance immunosuppression included tacrolimus (target trough 7–10 ng/mL), mycophenolate sodium, and a prednisone taper. The donor’s social history, including sexual risk factors, was unknown. Recipient serologies were notable for cytomegalovirus (CMV) seronegative status (R−) with a CMV-seropositive donor (D+). She completed 6 months of valganciclovir prophylaxis post-transplant. This was followed by serial CMV PCR surveillance every 2 weeks for 3 months after completion of prophylaxis, in accordance with institutional protocol. Both donor and recipient were Epstein–Barr virus (EBV) seropositive. On post-transplant day 6, donor microbiologic results demonstrated *Candida albicans* growth in one of five blood culture sets. Additional donor cultures, including urine and respiratory specimens, also yielded *Candida albicans*, reported as pan-susceptible. These cultures had initially been reported as negative, with delayed growth identified on extended incubation. At the time these results became available, the recipient had already undergone transplantation and was clinically stable. Caspofungin was initiated with a 70 mg loading dose, followed by 50 mg daily. Concurrent recipient investigations demonstrated *Candida albicans* growth in blood cultures, obtained from both central and peripheral lines, as well as from intra-abdominal drain cultures. Follow-up blood cultures demonstrated clearance of candidemia. In parallel, screening for blood-borne pathogens was performed in accordance with institutional protocol for donors with increased infectious risk. Given the timing of these findings, concern for probable donor-derived candidemia was raised. Molecular typing to confirm clonal identity between the donor and recipient isolates was not performed at our center. A transesophageal echocardiogram demonstrated a 7 mm filamentous structure on the left ventricular aspect of the aortic valve, most consistent with a degenerative lesion; however, vegetation could not be excluded. A repeat transesophageal echocardiogram performed later in the admission demonstrated an unchanged appearance. A dilated funduscopic examination was performed and was unremarkable, with no evidence of chorioretinitis or endophthalmitis. Given the patient’s hemodynamic stability and high operative risk, cardiac surgical intervention was deferred. The patient was managed medically for native aortic valve *Candida* endocarditis with high-dose caspofungin, 150 mg daily, for 6 weeks, followed by lifelong suppressive therapy with fluconazole 200 mg orally daily. This suppressive dose, below the 400–800 mg/day generally recommended for *Candida* endocarditis, was selected to balance the patient’s renal allograft function against the fluconazole–tacrolimus interaction. The contralateral kidney recipient from the same donor underwent screening and microbiologic evaluation and received fluconazole prophylaxis. No evidence of donor-derived *Candida* infection was detected, including negative blood cultures, and no fungal infection developed during follow-up. During the same admission, the patient developed a line-associated *Serratia marcescens* bacteremia, which was treated with imipenem, 500 mg intravenously every 8 h, for 14 days. Following discharge, she developed BK viremia, which was monitored in the outpatient setting by the Infectious Diseases team, with reduction of immunosuppression, including adjustment of mycophenolate. Approximately 14 months post-transplant, she developed left-sided rhino-orbital symptoms that progressed multiple emergency department (ED) encounters over two weeks. At the first visit, she reported one day of left periorbital redness and swelling, with no orbital cellulitis on examination, and was discharged on doxycycline for 7 days for presumed periorbital cellulitis. At a second visit during that antibiotic course, she reported new left maxillary incisor pain with poor dentition, and antimicrobial therapy was not changed. At a third visit, she had worsening facial swelling with new left V2-distribution sensory loss and photophobia; the first sinus computed tomography (CT) demonstrated sinusitis with bony erosion but no orbital cellulitis, and she was discharged on amoxicillin–clavulanate with an intranasal corticosteroid and otolaryngology follow-up. At a fourth visit, her symptoms persisted with a reassuring repeat CT, and she was again discharged. At the fifth visit, she presented with global worsening; repeat imaging demonstrated left orbital involvement with mild proptosis; complete opacification of the left maxillary sinus; partial opacification of the left ethmoid, frontal, and sphenoid sinuses; and erosive changes at the base of the left maxilla, consistent with pansinusitis and orbital cellulitis (Figure 1). Her glycemic control was poor at this time, with a hemoglobin A1c of 8.3% and no evidence of diabetic ketoacidosis. During the sinusitis workup, serum (1,3)-β-D-glucan and galactomannan were not present. She underwent endoscopic sinus surgery with debridement of the left maxillary sinus and orbital decompression. The next day, new signs of necrosis of the hard palate occurred, necessitating additional procedures included concha bullosa resection, polypectomy, maxillary antrostomy, ethmoidectomy, sphenoidotomy, and multiple biopsies. Intraoperatively, copious polypoid tissue and dark gray-brown necrotic material was seen. Specimens were sent for bacterial cultures and fungal staining. Empiric antibacterial therapy with intravenous vancomycin (20 mg/kg) and piperacillin–tazobactam (4.5 g every 6 h) had been initiated for a presumed bacterial odontogenic source; intravenous liposomal amphotericin B (L-AmB, 5 mg/kg) was added post-operatively given radiological and intra-operative findings suggestive of a fungal infection. MRI obtained following the initial sinonasal surgery demonstrated persistent inflammatory changes in the left post-septal orbital space with mild enlargement of the extraocular muscles (Figure 2a), diffusion restriction in the adjacent gingiva consistent with an abscess (Figure 2b), diffuse left preseptal periorbital cellulitis and facial myositis with subtle enhancement of the left cavernous sinus and pachymeninges (Figure 3a), and abnormal clival signal with nasopharyngeal thickening concerning for early acute osteomyelitis (Figure 3b). Histopathologic examination demonstrated invasive fungal elements consistent with mucormycosis, characterized by broad, ribbon-like, pauciseptate hyphae. The initial intraoperative fungal culture did not grow an organism despite fungal elements on microscopy; a repeat tissue culture subsequently grew *Rhizopus oryzae*. Antifungal susceptibility testing was not performed on the *Rhizopus* isolate, and standardized clinical breakpoints for *Mucorales* are not established. There was no evidence of disseminated disease. Serial blood cultures remained negative with no growth, and cross-sectional imaging—including CT of the chest and abdomen—did not demonstrate any features suggestive of dissemination. Antifungal therapy with liposomal amphotericin B was continued for approximately 10 to 11 weeks in conjunction with surgical debridement. This course was subsequently interrupted when the patient elected to pause active antifungal and surgical therapy during a period of care at an outside institution; she was also off suppressive fluconazole during this interval, the exact duration of which is not precisely documented in the available outside records. Following clinical reassessment and stabilization, oral antifungal therapy with isavuconazole was initiated, at a dose of 200 mg daily, and continued as prolonged consolidation therapy. All immunosuppressive agents were discontinued, resulting in graft failure and return to hemodialysis. She was subsequently reassessed for candidacy for kidney re-transplantation. On re-evaluation by Oral and Maxillofacial Surgery, she was found to have a persistent oroantral communication involving the left maxilla, likely representing sequelae of prior invasive infection. Examination demonstrated extensive dental disease requiring complete dental clearance prior to prosthetic rehabilitation. Plans are in place for staged dental extractions under general anesthesia, followed by consideration of maxillofacial reconstruction. In light of her prior invasive mucormycosis, antifungal prophylaxis with isavuconazole 200 mg orally for at least 12 months in the peri-transplant period has been recommended. However, access to isavuconazole has been intermittently limited due to drug coverage issues. She is currently maintained on fluconazole 200 mg orally daily for suppressive therapy of *Candida albicans* native aortic valve endocarditis. The plan is to transition to isavuconazole approximately two weeks prior to transplantation and continue for at least one year post-transplant. The patient currently remains dialysis-dependent and is undergoing intermittent hemodialysis. She is being considered for kidney retransplantation and remains active on the transplant waitlist pending suitability and timing of retransplant. In this peri-retransplant period, isavuconazole is intended to serve a dual purpose: secondary antifungal prophylaxis against mucormycosis recurrence and replacing fluconazole, chronic suppressive therapy for *Candida albicans* native-valve endocarditis with a single oral agent. The complete clinical course, including the donor-derived candidemia, subsequent invasive mucormycosis, antifungal treatment, current clinical status, and planned peri-retransplantation strategy, is summarized in Figure 4.

## 3. Discussion

We report a case of probable donor-derived *Candida albicans* endocarditis in a renal transplant recipient whose clinical course was further complicated by subsequent invasive rhino-orbital mucormycosis in the setting of prolonged immunosuppression and antifungal exposure lacking activity against *Mucorales* species. This case highlights the complex interplay between donor-derived infections (DDIs), antifungal exposure and breakthrough mold infections in solid organ transplant (SOT) recipients. DDIs in SOT recipients are uncommon, with reported incidence ranging from approximately 0.18% to 0.5%, yet they remain a significant clinical challenge due to their associated morbidity and mortality [1,2]. While bacterial and viral pathogens predominate, fungal DDIs, particularly those due to *Candida* species, are less frequent but are associated with disproportionately high mortality [3,4]. Despite advances in donor screening, transmission may occur when microbiologic results are delayed or initially negative, as seen in this case [2,5]. In this patient, the temporal association and species concordance between the donor and recipient *Candida albicans* isolates are consistent with donor-derived transmission. However, in the absence of strain typing (whole-genome sequencing, multilocus sequence typing, or pulsed-field gel electrophoresis), clonal identity cannot be established, and susceptibility concordance (the “pan-susceptible” donor isolates) reflects only shared drug activity rather than a shared strain. At least two alternative explanations therefore remain formally unexcluded: nosocomial acquisition during the peri-transplant period and endogenous translocation from the recipient’s own gastrointestinal or mucosal flora, the latter particularly relevant given the concurrent intra-abdominal drain cultures. We therefore classify the transmission as probable, rather than proven, donor-derived and regard this diagnostic uncertainty as a limitation of the report [1,2,3].

*Candida* endocarditis is a rare but severe manifestation of invasive candidiasis. Guidelines recommend combined surgical and systemic antifungal therapy when feasible, since medical therapy alone is associated with high relapse and mortality; when surgery is not possible, as in transplant recipients with prohibitive operative risk, prolonged antifungal therapy with consideration of lifelong suppression is recommended, typically a lipid formulation of amphotericin B or a high-dose echinocandin followed by step-down to an oral azole [6]. Observational cohorts confirm that outcomes remain suboptimal, that antifungal therapy alone is inferior to combined medical–surgical management, and that *Candida* species predominate in transplant-associated fungal endocarditis, in which prolonged non-surgical treatment is often the only option [7,8,9]. In our patient, medical management with an echinocandin followed by fluconazole suppression was selected on this basis. Application of the 2023 Duke–ISCVID criteria to this case merits comment. Because no intracardiac prosthetic material was present, *Candida albicans* does not qualify as a “typical” microorganism for infective endocarditis in this native-valve setting; the microbiological major criterion was instead satisfied by four different sets of positive cultures from multiple independent sites (central lines and peripheral blood). Together with the echocardiographic major criterion and a minor criterion (documented fever ≥ 38 °C), the case fulfilled two major criteria and one minor criterion, definite infective endocarditis [10]. Nonetheless, important diagnostic uncertainties remain. The 7 mm filamentous lesion is considerably smaller and less characteristic than vegetations typically reported in *Candida* endocarditis (median 13 mm, range 4–30 mm, in the MYCENDO cohort; mean 19.4 mm in an independent series) [11]. Candidemia in this patient was low-grade and transient, and the Duke criteria—which is derived predominantly from bacterial endocarditis and not rigorously validated in fungal disease—may under-represent this burden; distinguishing small or degenerative valve changes from early fungal vegetations is a recognized limitation of echocardiography. A repeat echocardiogram was unchanged and did not exclude the diagnosis. [18F]FDG PET/CT was not performed; under the 2023 Duke–ISCVID framework it is incorporated as a major imaging criterion specifically for prosthetic material at least three months after implantation, so its incremental value in native-valve disease is less established, although it may be a reasonable adjunct for baseline and longitudinal assessment in medically managed, non-surgical patients. Given documented candidemia, immunosuppression, and prohibitive surgical risk, treating this patient for endocarditis was clinically justified, although we acknowledge these limitations.

The subsequent development of invasive mucormycosis in this patient highlights the cumulative infectious risk in transplant recipients exposed to prolonged immunosuppression and antifungal agents without mold activity. Mucormycosis is an aggressive angioinvasive fungal infection with high mortality, particularly in patients with diabetes mellitus, prior antimicrobial exposure, and impaired host immunity [12,13,14,15]. SOT recipients represent a well-recognized high-risk population, with risk further amplified by T-cell-depleting induction therapies such as anti-thymocyte globulin and ongoing maintenance immunosuppression [16]. Importantly, prior antifungal exposure is often invoked in the emergence of breakthrough mold infections, but the mechanisms differ substantially among agents and warrant careful distinction. Fluconazole lacks activity against *Mucorales*; critically, however, it also lacks activity against *Aspergillus* and other filamentous molds, and therefore does not create the mold-selective ecological pressure attributed to voriconazole, which suppresses susceptible molds while sparing intrinsically resistant *Mucorales* and has additionally been shown experimentally to enhance the germination, angioinvasion, and virulence of *Rhizopus oryzae* in fly and murine models [17,18]. Caspofungin, which this patient also received, has likewise been associated with breakthrough mucormycosis in solid organ transplant recipients [19], although this association is less well characterized mechanistically and is not thought to enhance *Mucorales* virulence directly. Weighing these factors, we consider the most plausible dominant contributors in this patient to be poorly controlled diabetes (HbA1c 8.3%) and cumulative T-cell-depleting and triple-agent immunosuppression (rATG induction with tacrolimus, mycophenolate, and prednisone), both well-established independent risk factors for mucormycosis [12,15,16], with prior fluconazole and caspofungin exposure representing contributory rather than primary factors.

In our patient, invasive fungal disease was confirmed only after five ED encounters over approximately two weeks, during which two sequential empiric antibacterial regimens were prescribed while orbital involvement and tissue necrosis developed. Turning a critical eye to our own diagnostic trajectory, several factors could plausibly have shortened this interval. First, in a diabetic kidney transplant recipient already receiving suppressive fluconazole, an agent without *Mucorales* activity, antibiotic-refractory sinusitis should itself have triggered early tissue biopsy; in this population, the threshold for biopsy should be antibiotic non-response alone, not contingent on a supportive biomarker. Second, the serum (1,3)-β-D-glucan and galactomannan obtained later during the workup were negative, but neither assay detects *Mucorales* [12]; a negative fungal biomarker in this setting must not be allowed to lower clinical suspicion for mold disease or to defer biopsy. Third, earlier involvement of infectious diseases and otolaryngology with cross-sectional imaging at the point of antibiotic failure would likely have accelerated diagnosis and treatment. This matters because delayed initiation of active antifungal therapy is directly associated with increased mortality in mucormycosis; in a landmark hematology cohort, delaying amphotericin B-based therapy beyond six days increased 12-week mortality from 48.6% to 82.9% [17]. Molecular diagnostics such as *Mucorales*-specific PCR on serum or tissue can shorten the diagnostic interval where an initial culture fails to grow the organism [20]; however, this assay was not available at the institution—an infrastructure gap that is itself relevant for centers weighing whether to develop this capability. We therefore regard this potentially avoidable diagnostic delay as a central teaching point of the case.

A recent systematic review by Emanuele Palomba et al., including 183 cases of mucormycosis in solid organ transplant recipients, demonstrated that kidney transplant recipients accounted for approximately half of all cases. Notably, 63.7% of infections occurred within the first 100 days post-transplant, although late infections were also observed, with 20.6% occurring more than one year after transplantation. The most common clinical presentations were pulmonary and rhino-orbital-cerebral disease, and outcomes remained poor despite modern antifungal therapy and surgical management, with reported 90-day and 1-year mortality rates of 36.3% and 63.4%, respectively [21]. In our patient, the development of rhino-orbital mucormycosis approximately 14 months following renal transplantation aligns with the subset of late-onset infections described in this cohort. While early post-transplant disease predominates, these findings highlight that mucormycosis remains an important late complication in transplant recipients. The renal transplant status and rhino-orbital pattern of disease in this case further mirror the predominant organ type and clinical presentations reported in this population. Optimal management of mucormycosis requires a multimodal approach, including early initiation of active antifungal therapy, aggressive surgical debridement, and reversal of underlying immunosuppression when possible. Liposomal amphotericin B remains the first-line therapy; however, nephrotoxicity frequently limits its use, particularly in transplant recipients or those with pre-existing renal dysfunction [22,23]. Isavuconazole has emerged as an effective alternative for the treatment of mucormycosis, particularly in patients unable to tolerate amphotericin B. Evidence supporting its use comes from the VITAL trial conducted by Marty et al., a multicenter, open-label study evaluating isavuconazole in patients with invasive fungal disease, including mucormycosis, which demonstrated comparable efficacy to amphotericin-based regimens with improved tolerability and safety [24]. Beyond this, accumulating evidence from systematic reviews and real-world studies has further supported its role across diverse clinical settings. A recent systematic review by Gunathilaka et al. demonstrated consistent effectiveness of isavuconazole in both primary and salvage therapy, with favorable safety outcomes, particularly in patients requiring prolonged treatment or with underlying renal dysfunction [25]. Compared with amphotericin B, isavuconazole offers several advantages, including lack of nephrotoxicity, predictable pharmacokinetics, and availability in both intravenous and oral formulations, making it particularly suitable for long-term management in transplant recipients. In addition, unlike fluconazole, isavuconazole has reliable activity against *Mucorales* species, supported by in vitro susceptibility data from contemporary global isolate collections [26,27]. In our case, transition to isavuconazole following amphotericin-associated nephrotoxicity aligns with this evolving evidence base and is consistent with current evidence. The choice of isavuconazole rather than posaconazole for consolidation and secondary prophylaxis also merits comment. Although posaconazole shows numerically greater in vitro potency against *Rhizopus* spp. than isavuconazole (MIC50/90 0.5/8 versus 1/>8 mg/L) [26], the 2019 ECMM/MSGERC global guideline assigns the two agents equivalent recommendation strength at each treatment stage, without a stated preference between them [22]. Because posaconazole is a strong CYP3A4 inhibitor requiring substantially larger tacrolimus dose reductions than the moderate inhibitor isavuconazole (approximately 53% versus 21% in comparative transplant data) [28], and given this patient’s retransplant candidacy and need for predictable tacrolimus dosing, we judged isavuconazole’s more favorable drug-interaction and safety profile to outweigh its comparatively lower in vitro potency against *Rhizopus* spp. in this specific context. Importantly, Reduction or discontinuation of immunosuppression is a critical component of management but carries a substantial risk of graft dysfunction or loss [27].

In this patient, cessation of immunosuppression was required to control infection, ultimately resulting in graft failure and return to hemodialysis, illustrating the well-described tension between infection control and graft preservation in transplant populations. Prior invasive mucormycosis has traditionally been viewed as a major concern in transplant candidates due to the risk of recurrence under renewed immunosuppression, and has often influenced decisions regarding re-transplantation in clinical practice. However, emerging clinical experience and the transplant infectious diseases literature suggest that re-transplantation may be feasible in carefully selected patients following adequate infection control, prolonged antifungal therapy, and demonstration of clinical and radiologic stability [29]. Importantly, recurrence of mucormycosis has been reported even after apparent clinical resolution, including in transplant recipients, underscoring the need for cautious patient selection and prolonged monitoring [29,30,31,32]. Secondary antifungal prophylaxis is critical in this setting. Current international guidelines emphasize the importance of prolonged antifungal therapy and individualized prevention strategies in patients undergoing renewed immunosuppression, particularly when residual risk factors persist [22]. Isavuconazole is increasingly favored due to its activity against *Mucorales* species, favorable safety profile, and availability in both intravenous and oral formulations, making it particularly suitable for long-term use in transplant recipients [24,25]. In our patient, planned peri-transplant isavuconazole reflects a deliberate dual-purpose strategy: secondary prophylaxis against mucormycosis recurrence and, replacing fluconazole, chronic suppressive therapy for *Candida albicans* native-valve endocarditis using a single oral agent. We consider this one of the more original and clinically relevant aspects of the case, and we emphasize explicitly that it is off-label and supported only by isolated case reports. Reported experience with isavuconazole as a long-term suppressive therapy for *Candida* endocarditis includes AngioVac-assisted management of *Candida tropicalis* endocarditis with subsequent long-term isavuconazole suppression [33], and isavuconazole suppression of *Candida albicans* endocarditis in a patient with irreplaceable prosthetic cardiac material; notably, the latter patient developed breakthrough candidemia after approximately two months, requiring temporary reversion to amphotericin B and flucytosine before isavuconazole could be re-established [34]. Because isavuconazole generally has higher MICs against *Candida* species than fluconazole or the echinocandins, breakthrough is a genuine risk during prolonged suppression, and we therefore recommend close clinical and microbiological surveillance, including consideration of periodic echocardiographic follow-up, during long-term isavuconazole use in this patient.

## Data Availability

The original contributions presented in this study are included in the article. Further inquiries can be directed to the corresponding author.
